# Integrated Bulk Transcriptomic and Single-Cell Analyses Reveal Extracellular-Matrix Remodeling and Tubular Metabolic Failure in ADPKD Progression

**DOI:** 10.3390/biology15211939

**Published:** 2026-10-25

**Authors:** Changan Chen, Lingzhi Lei, Yanfang Xu, Zhimin Chen

**Affiliations:** 1 The First Clinical Medical College of Fujian Medical University, The First Affiliated Hospital of Fujian Medical University, Fuzhou 350005, China; 2 Department of Nephrology, Blood Purification Research Center, The First Affiliated Hospital, Fujian Medical University, Fuzhou, 350005, China; 3 Research Center for Metabolic Chronic Kidney Disease, The First Affiliated Hospital, Fujian Medical University, Fuzhou 350005, China; 4 Department of Nephrology, National Regional Medical Center, Binhai Campus of the First Affiliated Hospital, Fujian Medical University, Fuzhou 350212, China; 5 Central Laboratory, The First Affiliated Hospital, Fujian Medical University, Fuzhou 350005, China

**Keywords:** ADPKD, single-cell RNA sequencing, extracellular matrix, tubular metabolism, immune microenvironment

## Abstract

Background: Autosomal dominant polycystic kidney disease (ADPKD) progression involves cyst expansion, interstitial remodeling, and progressive loss of renal function, but the cell-type-resolved programs linking cyst burden to tissue remodeling remain incompletely defined. Methods: We integrated the cyst-size-stratified human bulk transcriptomic dataset GSE7869 with the time-resolved *Pkd1* single-cell dataset GSE268494. Differentially expressed genes were identified across small-, middle- and large-cyst groups, modeled by Mfuzz temporal clustering, prioritized by protein–protein interaction topology and functional enrichment, and further evaluated by immune deconvolution, single-cell mapping, AUCell pathway scoring and CellPhoneDB ligand–receptor inference. Results: We identified 3842 shared differentially expressed genes and two dominant monotonic modules. The progressively upregulated module was enriched for extracellular-matrix organization, adhesion, and fibrosis-associated genes, including *COL1A1, COL5A2, FBN1, BGN, LAMC1, HSPG2, MMP2, LOX,* and *ITGA5*. The progressively downregulated module highlighted tubular transport, proton transport, and mitochondrial/metabolic genes, including *SLC12A3, AQP2, ATP5A1, ATP6V0C, ATP6V1B1, NDUFV1, REN*, and *AGT*. Single-cell analyses localized the matrix module mainly to fibroblast/stromal compartments and the transport/metabolic module to segment-specific tubular epithelial populations. Immune deconvolution and ligand–receptor inference further implicated macrophage-associated remodeling and TGFB1-, CD47-, CD200-, PECAM1- and TNF-superfamily-related immune-parenchymal communication. Conclusions: ADPKD progression is associated with coordinated ECM/immune remodeling and tubular metabolic failure, providing candidate markers and communication axes for future spatial and functional validation.

## Simple Summary

Polycystic kidney disease is an inherited kidney disease in which fluid-filled cysts keep growing in the kidneys. As cysts enlarge, kidney tissue becomes scarred and kidney function declines, but it is still unclear how different kidney cells drive the disease. We wanted to know which genes become more active and which become less active as the disease worsens. To find out, we grouped human kidney samples by cyst size and combined them with cell-by-cell information from the kidneys of diseased mice. We found two opposite trends. One trend increased steadily and came mainly from supporting tissue cells; it involved genes that build scar-like tissue. The other trend decreased steadily and came mainly from kidney tube cells that filter and transport; it involved genes needed for filtering, transport, and energy production. Immune cells called macrophages also appeared to help drive scarring and communicate with kidney tissue. Together, these results suggest that worsening disease involves scarring, immune activity, and energy failure in kidney cells. They also point to possible clues and treatment targets for monitoring or slowing polycystic kidney disease.

## 1. Introduction

Autosomal dominant polycystic kidney disease (ADPKD) is the most common inherited cystic kidney disease and a major monogenic cause of kidney failure. Most cases are caused by pathogenic variants in *PKD1* or *PKD2*, which encode the polycystin complex involved in primary-cilium signaling, calcium homeostasis, epithelial polarity, and tubular architecture. Clinically, ADPKD is characterized by progressive bilateral renal cyst expansion, kidney enlargement, hypertension, pain, infection, nephrolithiasis, and gradual loss of renal function. Although genotype is a major determinant of prognosis, patients with similar genetic backgrounds can show markedly different rates of cyst growth, interstitial fibrosis, and kidney-function decline, indicating that secondary tissue programs substantially shape disease trajectory [1,2].

Current therapeutic management remains clinically important but biologically incomplete. Tolvaptan can slow total kidney-volume expansion and estimated glomerular filtration-rate decline in selected patients, and supportive management of blood pressure, cardiovascular risk and kidney complications remains essential [1,2]. However, available therapies do not fully prevent progression to kidney failure, and treatment selection still relies heavily on clinical risk stratification rather than on molecular staging. This creates a need to define disease-associated molecular modules that reflect cyst burden, stromal remodeling and tubular functional impairment, and that could eventually support biomarker discovery or mechanism-guided intervention.

The classical epithelial model of ADPKD emphasizes altered calcium and cyclic AMP signaling, cyst epithelial proliferation, planar-cell-polarity defects and transepithelial fluid secretion [1]. Recent single-cell and multiomic studies have broadened this framework by identifying cyst-lining epithelial states, failed-repair tubular programs, proinflammatory fibroblast populations, vascular remodeling, and altered chromatin accessibility [3,4]. These observations support a multicellular view in which tubular epithelial stress, stromal remodeling, vascular changes and immune activation reinforce one another over time, rather than acting as isolated pathological compartments.

Immune and extracellular-matrix (ECM) remodeling are particularly relevant to ADPKD progression. Macrophage polarization, inflammatory cytokine signaling, complement activation, T-cell involvement and immune checkpoint pathways have been implicated in cyst growth and fibrosis [5,6,7]. ECM dynamics, including collagen deposition, basement-membrane remodeling, matrix crosslinking and metalloprotease activity, may actively alter tissue stiffness, epithelial polarity, integrin signaling, and mechanotransduction rather than simply reflect end-stage scar formation [8,9]. In parallel, metabolic reprogramming and mitochondrial dysfunction have emerged as candidate mechanisms linking epithelial stress to cyst expansion, impaired transport activity, and susceptibility to maladaptive repair [10,11,12,13,14,15,16,17].

Bulk transcriptomic studies can capture disease-stage-associated signals, but they often obscure whether the dominant signals arise from epithelial cell-state changes, stromal activation, immune infiltration, or altered cellular composition. Conversely, single-cell datasets provide cellular resolution but may be limited by model system, sampling depth, and the difficulty of directly aligning experimental time points with human cyst burden [18]. Integrating cyst-size-stratified human transcriptomic data with a time-resolved single-cell reference therefore provides a practical strategy for separating shared progression programs from cell-type-specific mechanisms.

Overall, two key gaps remain. First, interpretable transcriptional modules that link cyst burden, stromal remodeling, and tubular dysfunction in human ADPKD are still lacking. Second, it remains unclear whether cyst-size-associated transcriptional changes in human ADPKD reflect shared progression programs or compartment-specific responses. In this study, we integrated cyst-size-stratified human bulk transcriptomic data with a *Pkd1* time-course single-cell dataset to reconstruct stage-associated molecular programs in ADPKD. We first identified differentially expressed genes (DEGs) across small-, middle-, and large-cyst groups, used Mfuzz to classify temporal expression patterns, and then characterized candidate modules by network topology, enrichment analysis, immune deconvolution, single-cell localization, pathway scoring, and ligand–receptor inference. The aim was to determine whether cyst-size-associated bulk transcriptional trends could be assigned to biologically interpretable renal cell compartments and used to nominate candidate mechanisms for future spatial and functional validation.

## 2. Materials and Methods

### 2.1. Public Datasets and Preprocessing

Human ADPKD microarray data (GSE7869) were downloaded from the GEO database using GEOquery (v2.66.0) [19,20]. We selected control, small-cyst, medium-cyst, and large-cyst groups from this human ADPKD renal tissue dataset for stage-resolved transcriptomic analysis (normal control: *n* = 3; small cysts [<1 mL]: *n* = 5; medium cysts [10–25 mL]: *n* = 5; large cysts [>50 mL]: *n* = 3; total *n* = 16). Raw probe-level data were background-corrected and quantile-normalized. Probes mapping to multiple genes were removed, and multiple probes targeting the same gene were averaged. Non-logarithmic expression values were log2-transformed for standardized downstream analysis.

### 2.2. Differential Expression Analysis

Stage-related DEGs were identified using limma (v3.54.0) in R (v4.2) [21]. We performed independent comparisons of small-, middle-, and large-cyst groups versus control tissues. Linear models were fitted with lmFit, and statistical moderation was conducted via empirical Bayes estimation. DEGs were defined by adjusted *p* < 0.05 and |log2FC| > 1, with Benjamini–Hochberg correction for multiple testing. Volcano plots and Venn diagrams were generated using ggplot2(v3.4.2), VennDiagram(v1.7.3), and ggVennDiagram(v1.2.3) to visualize DEG distribution and overlap across stages.

### 2.3. Mfuzz Temporal Clustering

To characterize dynamic gene expression patterns during cyst progression, overlapping DEGs across all cyst stages were subjected to soft clustering using Mfuzz (v2.58.0). Gene expression values were ordered by disease progression (control, small, middle, large cyst) and standardized prior to clustering. The fuzzification parameter m was computationally estimated, and six clusters were set to capture major increasing, decreasing, and non-monotonic expression trends. Genes with high membership in progressive up- and downregulated clusters were retained for subsequent network analysis.

### 2.4. PPI Network Construction and Hub-Gene Prioritization

Genes from the progressively upregulated and downregulated temporal clusters were submitted to the STRING database (v11.5) with Homo sapiens as the reference organism [22]. Interactions with a combined score > 0.4 were retained. The resulting networks were imported into Cytoscape (v3.10.3). The cytoHubba plugin was used to calculate node centrality by 13 algorithms, including MCC, MNC, Degree, Closeness, Betweenness, Stress, EcCentricity, BottleNeck, EPC, Radiality, ClusteringCoefficient, DMNC, and Maximal Clique-related scores when available. For each algorithm, the top 200 nodes were retained for comparison, and Z-score-normalized centrality matrices were used to rank candidate hub genes.

### 2.5. Functional Enrichment Analysis

GO and KEGG enrichment analyses were performed using clusterProfiler (v4.6.0) and org.Hs.eg.db (v3.16.0). Gene symbols were converted to Entrez IDs for hypergeometric statistical testing. Enriched terms with adjusted *p* < 0.05 were considered significant. Redundant GO terms were simplified by semantic similarity. Enrichment results were visualized via bubble plots using enrichplot(v1.18.2) and ggplot2 according to gene ratio, statistical significance, and gene count.

### 2.6. Immune Infiltration and Correlation Analysis

Immune and stromal cell abundance in bulk ADPKD samples was estimated using xCell (v1.1.0). Cell types with implausible renal representation or low variability were excluded. Stage-dependent infiltration differences were assessed using Wilcoxon rank-sum or Kruskal–Wallis tests. Spearman correlation analysis was applied to evaluate associations between hub gene expression and immune cell scores, with correlation heatmaps generated using pheatmap(v1.0.12) and Complex Heatmap(v2.14.0).

### 2.7. Single-Cell RNA Sequencing Processing and Cell-Type Annotation

A *Pkd1* time-course single-cell dataset (GSE268494) was used for cell-type-resolved validation [4]. This dataset comprises 16 samples across three time points (P66, P100, and P130), with each time point including both control and *Pkd1* groups (P66: *n* = 2 control, *n* = 2 *Pkd1*; P100: *n* = 3 control, *n* = 3 *Pkd1*; P130: *n* = 3 control, *n* = 3 *Pkd1*; total *n* = 16). Single-cell RNA analysis was performed in R using Seurat (v4.2.0) [23]. Ambient RNA contamination was corrected with SoupX (v1.6.1) [24]. The following parameters were used to remove low-quality cells: (1) exclude cells expressing ≤500 or ≥4000 genes/cell; (2) exclude cells expressing ≤500 or ≥15000 unique molecular identifier per cell (UMIs/cell); (3) exclude cells with high cell complexity (log10GenesPerUMI) ≤0.8; (4) due to the fact that renal tubular epithelial cells in the kidney are very energetically involved in active transport, which requires a large number of mitochondria to provide energy. We excluded cells with >30% mitochondrial ratio; (5) doublets using the DoubletFinder (Version 2.0.3) package [25]; and (6) retained genes expressed in at least 10 cells. Data were normalized, and highly variable genes were selected for PCA dimensionality reduction. Batch effects were corrected via Harmony(v0.1.1). Cells were clustered at optimized resolution (0.5–1.5) and annotated using canonical renal cell markers for major parenchymal, stromal, and immune populations.

### 2.8. Candidate-Gene Mapping and AUCell Pathway Scoring

Core candidate genes from clustering and network analysis were mapped to single-cell clusters and visualized by dot plots to display cell-type-specific expression patterns. To quantify stage-specific pathway activity at single-cell resolution, AUCell (v1.20.2)was used to calculate pathway scores based on MSigDB(v7.5.1) gene sets. Pathway activities were compared across early (P66), middle (P100), and late *Pkd1* disease stages.

### 2.9. Ligand–Receptor Communication and Statistical Analysis

Cell–cell communication was inferred using CellPhoneDB (v5.0.1) with normalized single-cell expression and cell annotation data [26]. Ligand–receptor interactions were evaluated via 1000 permutations, and interactions with *p* < 0.05 were considered significant. Analyses were performed separately for control and *Pkd1*-deficient groups. Results were visualized using ktplots (v2.0.0) or custom R scripts. Interactions involving B cells, T cells, myeloid cells, and renal parenchymal/stromal cells were prioritized for display.

### 2.10. Statistical Analysis

Continuous variables were compared using Wilcoxon rank-sum tests for two groups and Kruskal–Wallis tests for multiple groups. Correlations were assessed using Spearman coefficients. Multiple testing correction was performed using the Benjamini–Hochberg method. Two-sided *p* values < 0.05 were considered statistically significant.

## 3. Results

### 3.1. Cyst-Size Transcriptomes Reveal Stage-Associated Gene Dysregulation

**Figure 1:**
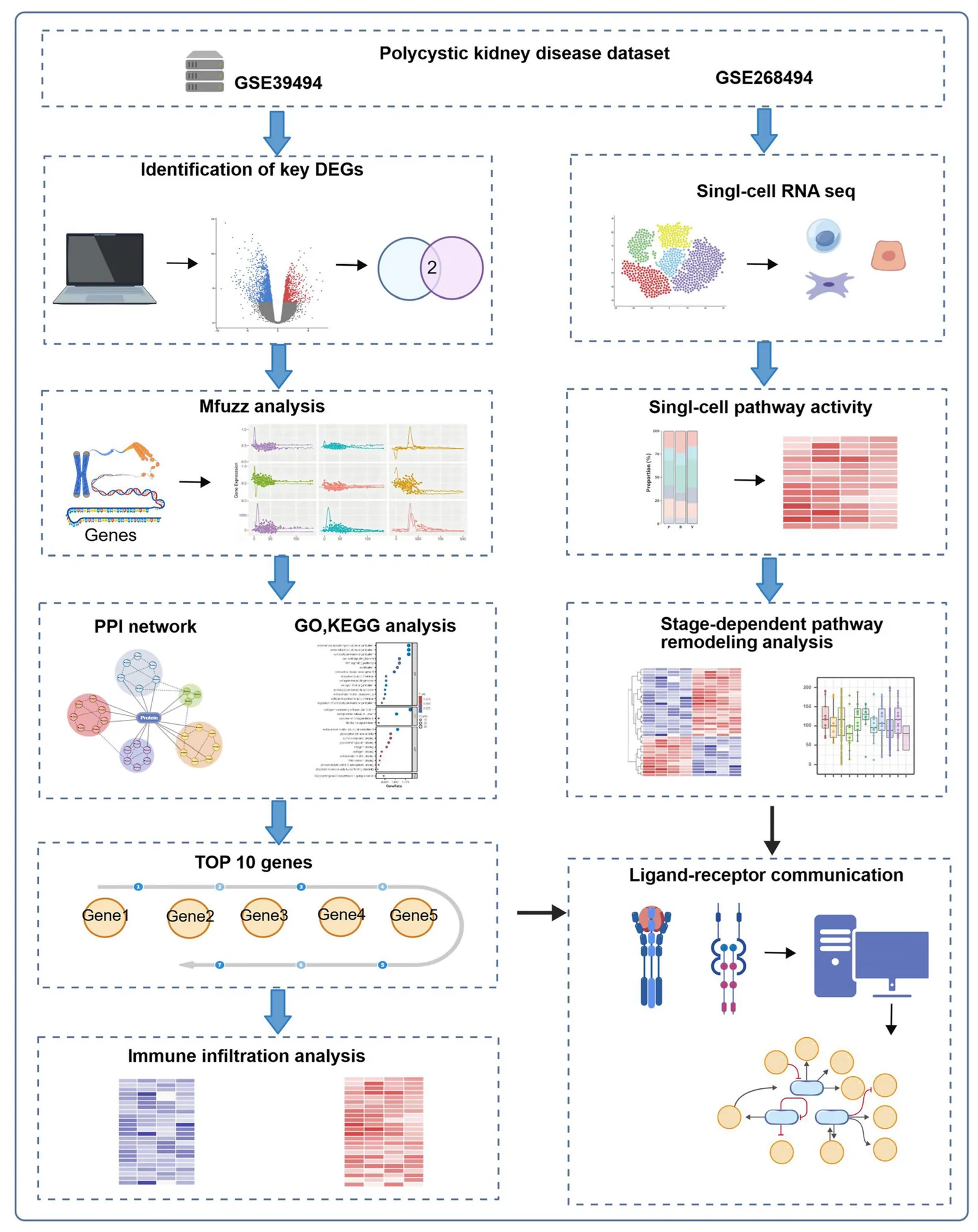
Analytical workflow.The study integrates human cyst-size-stratified bulk transcriptomics with mouse *Pkd1* single-cell validation. Bulk DEGs were identified from small-, middle-, and large-cyst comparisons, shared DEGs were modeled by Mfuzz, candidate temporal modules were prioritized by PPI topology and functional enrichment, and the resulting signatures were evaluated by immune deconvolution, single-cell mapping, AUCell pathway scoring, and ligand–receptor inference.

Transcriptomic profiling stratified by cyst size revealed progressive, stage-dependent transcriptional dysregulation in human ADPKD kidneys, with a defined analytical workflow integrating bulk discovery and multi-layer validation (Figure 1). This framework enables the prioritization of cyst-stage-associated transcriptional signals and subsequent attribution of these signals to discrete renal cell compartments. Differential expression analysis was performed by comparing small-, middle-, and large-cyst tissues against control tissues to capture early and late transcriptional alterations accompanying cyst expansion.

**Figure 2:**
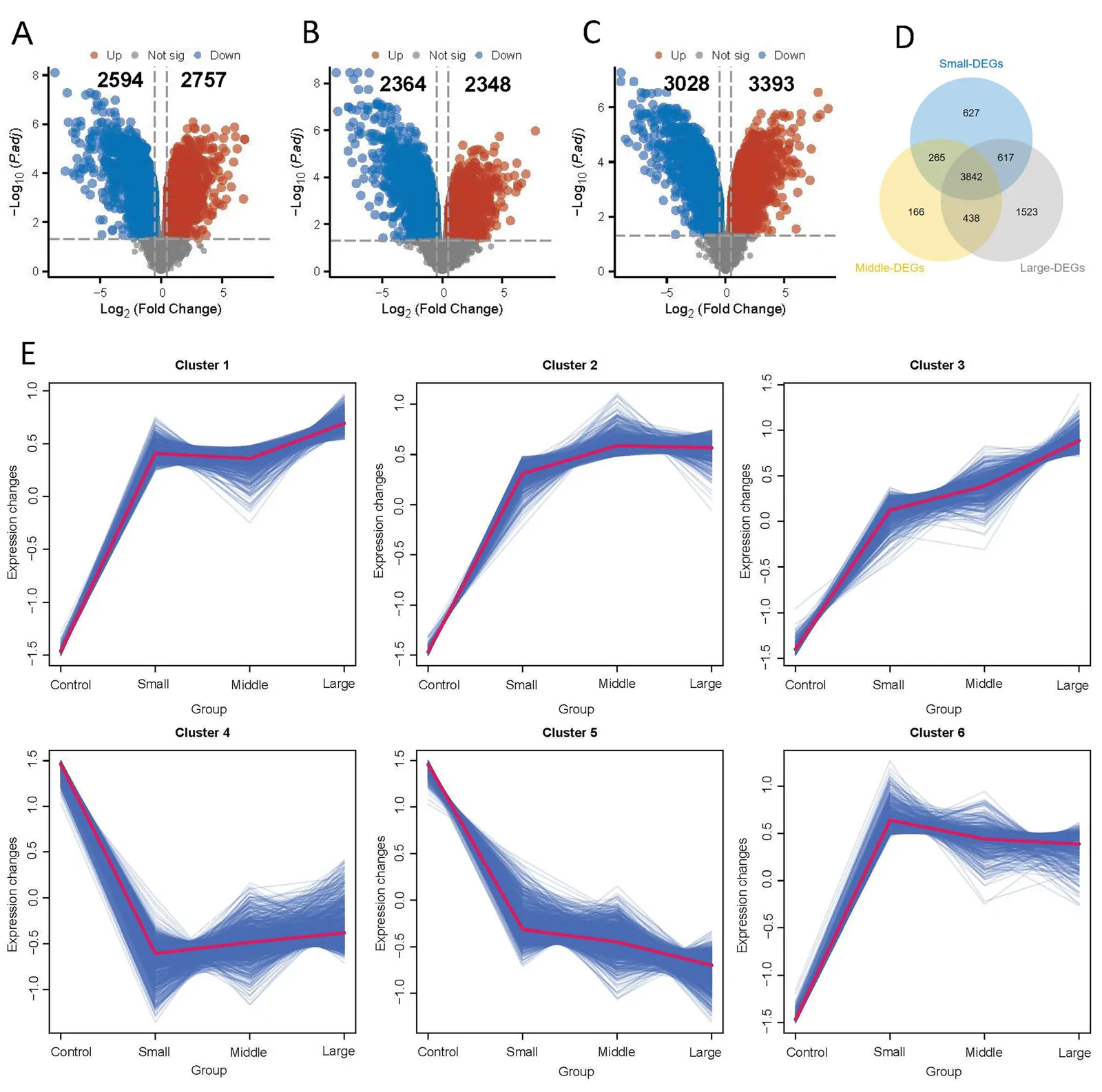
Differential expression and temporal trend analysis across ADPKD cyst-size stages. (**A**) Volcano plot showing DEGs in the small-cyst versus comparator comparison. Red points indicate upregulated genes, blue points indicate downregulated genes and gray points indicate genes that did not meet the significance threshold. (**B**) Volcano plot showing DEGs in the middle-cyst comparison. (**C**) Volcano plot showing DEGs in the large-cyst comparison. (**D**) Venn diagram showing the overlap among DEGs from the small-, middle-, and large-cyst comparisons; 3842 shared DEGs were retained for temporal modeling. (**E**) Mfuzz soft-clustering trends of shared DEGs across the control, small-cyst, middle-cyst and large-cyst states. Cluster 3 represents progressively upregulated genes during cyst enlargement, whereas Cluster 5 represents progressively downregulated genes.The blue lines represent the expression profiles of individual genes assigned to each cluster, while the thick red lines indicate the cluster centroids (average expression trends) for each of the six clusters.

Widespread transcriptomic perturbation was observed across all three cyst-size groups in dataset GSE7869 (Figure 2A–C). The small-cyst group identified 2594 upregulated and 2757 downregulated genes; the middle-cyst group identified 2364 upregulated and 2348 downregulated genes; and the large-cyst group identified 3028 upregulated and 3393 downregulated genes (Supplementary Table S1). These results suggest that ADPKD-associated transcriptional remodeling is already detectable in smaller cystic tissues and becomes broadly amplified in large cysts.

Intersection analysis identified 3842 shared DEGs across the three cyst-size comparisons (Figure 2D). This shared gene set was selected for temporal modeling, as it represents a consistent disease-associated signature rather than stage-restricted transcriptional events. Mfuzz soft clustering separated the shared DEGs into six temporal clusters ordered from control tissue to small, middle, and large cysts (Figure 2E). Cluster 3 showed progressive upregulation with cyst enlargement, while Cluster 5 showed progressive downregulation. These two monotonic clusters were prioritized as the core transcriptional programs for downstream mechanistic analyses.

### 3.2. Network and Enrichment Analyses Identify ECM and Tubular Homeostasis Modules

**Figure 3:**
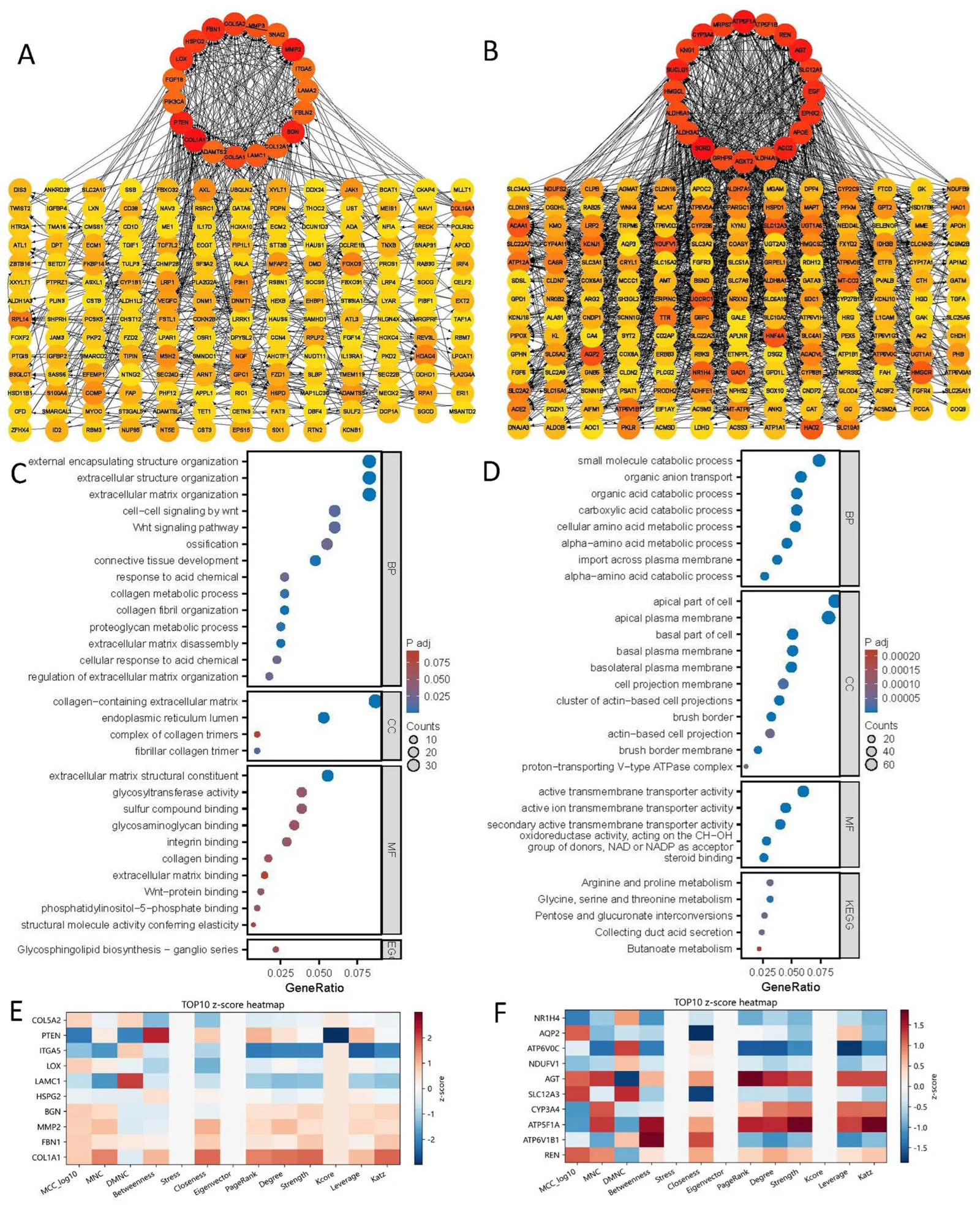
Network and functional characterization of Cluster 3 and Cluster 5 genes. (**A**,**B**) PPI network of progressively upregulated Cluster 3 genes (**A**) and progressively downregulated Cluster 5 genes (**B**) generated using STRING and visualized in Cytoscape. All nodes are set to identical size. Node color gradient represents gene degree (connectivity) within the PPI network: red nodes indicate hub genes with higher connectivity, while yellow nodes represent genes with lower connectivity. Edges indicate protein–protein interactions. (**C**) GO/KEGG enrichment analysis of Cluster 3, highlighting extracellular matrix organization, collagen-containing matrix, cell-substrate adhesion, glycosaminoglycan binding, and Wnt-related signaling. (**D**) GO/KEGG enrichment analysis of Cluster 5, highlighting solute transport, epithelial membrane compartments, proton transport, small-molecule catabolism, and mitochondrial/metabolic pathways. (**E**) Heatmap of top Cluster 3 hub genes ranked by multiple cytoHubba algorithms after Z-score normalization of topology scores. (**F**) Heatmap of top Cluster 5 hub genes ranked by the same multi-algorithm strategy.

PPI network construction and functional enrichment were performed separately for genes within Cluster 3 and Cluster 5. The Cluster 3 network contained highly connected ECM, adhesion, and fibrosis-associated genes including *COL1A1, COL5A2, FBN1, BGN, LAMC1, HSPG2, MMP2, LOX*, and *ITGA5* (Figure 3A). The concurrent enrichment of structural matrix proteins, basement membrane components, matrix-modifying enzymes, and adhesion molecules suggests that cyst expansion is accompanied by coordinated ECM remodeling instead of isolated induction of collagen genes.

Functional enrichment further supported this observation for Cluster 3 (Figure 3B). Upregulated genes were enriched in terms including extracellular matrix organization, extracellular structure organization, collagen-containing extracellular matrix, cell-substrate adhesion, glycosaminoglycan binding, Wnt signaling, and matrix remodeling terms. These categories connect structural remodeling with signaling processes that can influence epithelial–stromal crosstalk and tissue mechanics.

In contrast, the Cluster 5 network was enriched for genes related to epithelial transport, mitochondrial function, and nephron-segment identity (Figure 3C). High-priority genes included *SLC12A3, REN, NR1H4, NDUFV1, CYP3A4, ATP6V1B1, ATP6V0C, ATP5A1, AQP2*, and *AGT*. Enrichment analysis of Cluster 5 highlighted small-molecule catabolism, organic acid metabolism, apical and basolateral membrane compartments, ion transmembrane transport, proton transport, and mitochondrial/metabolic pathways (Figure 3D). Thus, the two major temporal modules captured opposing processes: progressive ECM and fibrotic activation and progressive loss of tubular transport/metabolic specialization.

Multi-algorithm ranking via cytoHubba further validated this functional dichotomy (Figure 3E,F). Cluster 3 hub ranking repeatedly prioritized ECM and adhesion genes including *COL5A2, ITGA5, LOX, LAMC1, HSPG2, BGN, MMP2, FBN1,* and *COL1A1*, whereas Cluster 5 hub ranking prioritized tubular transport and metabolic genes including *SLC12A3, REN, NDUFV1, ATP6V1B1, ATP6V0C, ATP5A1, AQP2*, and *AGT*. The convergence of temporal trend, network topology, and enrichment results indicates that ECM remodeling and tubular functional failure are dominant, coordinated features of ADPKD progression.

### 3.3. Immune Deconvolution Links Candidate Modules to Microenvironmental Remodeling

**Figure 4:**
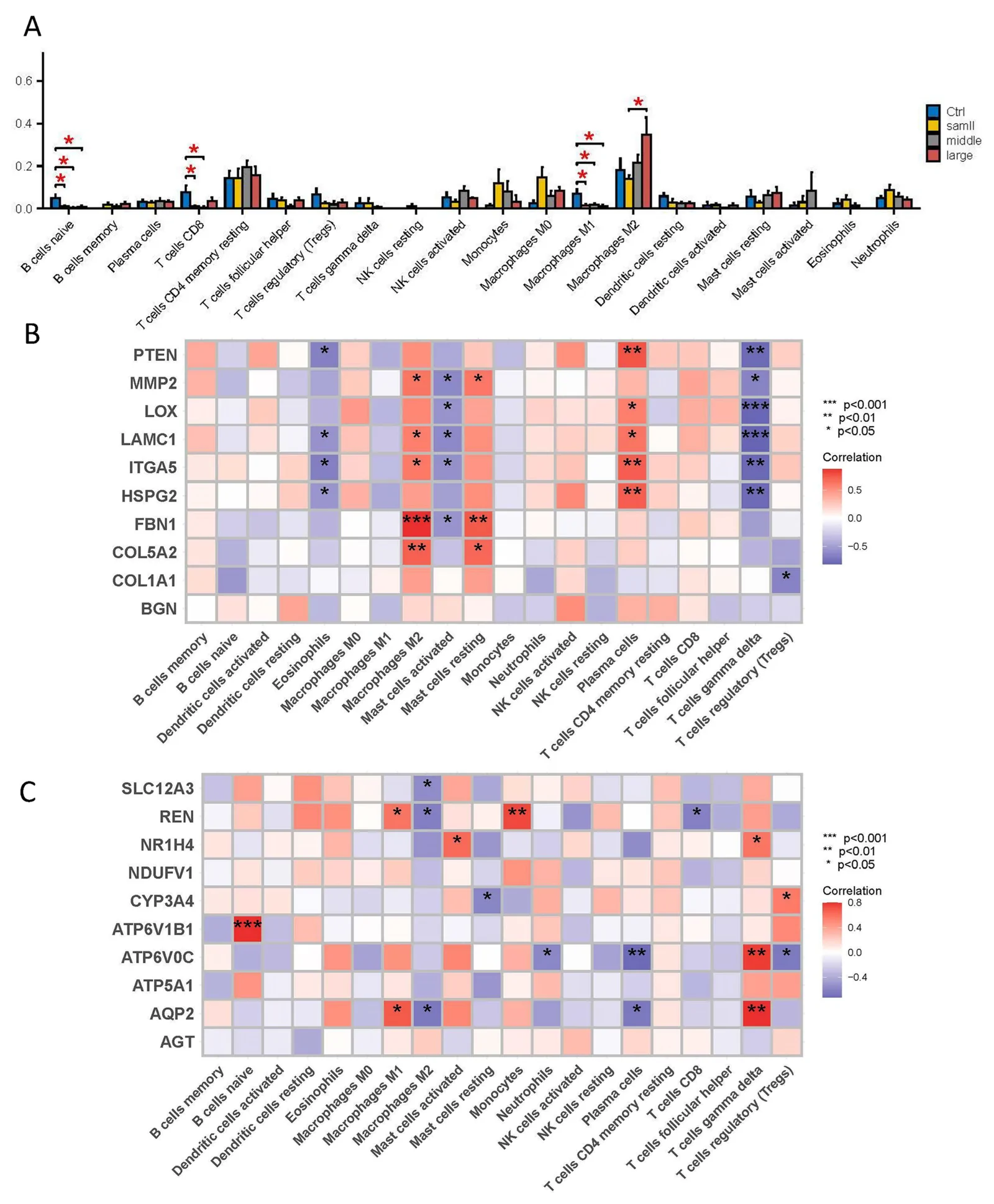
Immune infiltration and immune-correlation analysis in cyst-size-stratified bulk data. (**A**) xCell-inferred immune and stromal cell enrichment scores across control, small-cyst, middle-cyst, and large-cyst groups (* *p* < 0.05). (**B**) Correlation heatmap linking top Cluster 3 ECM-remodeling genes to inferred immune-cell scores; red indicates positive correlation and blue indicates negative correlation. (**C**) Correlation heatmap linking top Cluster 5 tubular transport/metabolic genes to inferred immune-cell scores. Asterisks denote statistical significance (* *p* < 0.05, ** *p* < 0.01 and *** *p* < 0.001).

Expression distributions of prioritized hub genes supported the temporal patterns detected by Mfuzz. ECM-associated genes increased progressively across cyst-size groups, while tubular transport and metabolic genes tended to decrease (Supplementary Figure S1). Immune deconvolution was performed to determine whether these transcriptional modules were accompanied by changes in immune and stromal cell abundance (Figure 4A). The predicted immune microenvironment differed across control, small-, middle-, and large-cyst groups, with macrophage, mast cell, and lymphocyte signatures exhibiting stage-dependent changes. Correlation analysis linked candidate genes to the inferred immune microenvironment (Figure 4B,C). Cluster 3 genes, especially *MMP2, LOX, LAMC1, ITGA5*, and *HSPG2*, showed positive correlations with macrophage- and mast-cell-associated signals including M2-like macrophage enrichment patterns. These associations suggest that the progressively increasing ECM module occurs within an immune-remodeled cystic tissue environment and may be coupled to macrophage- and fibroblast-associated remodeling.

Cluster 5 genes showed a different immune-correlation pattern, consistent with epithelial homeostasis loss in a changing tissue context. Transport and metabolic genes such as *AQP2, SLC12A3, ATP5A1*, and *ATP6V1B1* may decline because of both reduced differentiated epithelial function and altered cellular composition. Together, the immune deconvolution data provide a bridge between bulk temporal modules and the later single-cell communication analysis.

### 3.4. Single-Cell Mapping Resolves the Cellular Context of Bulk-Derived Modules

**Figure 5:**
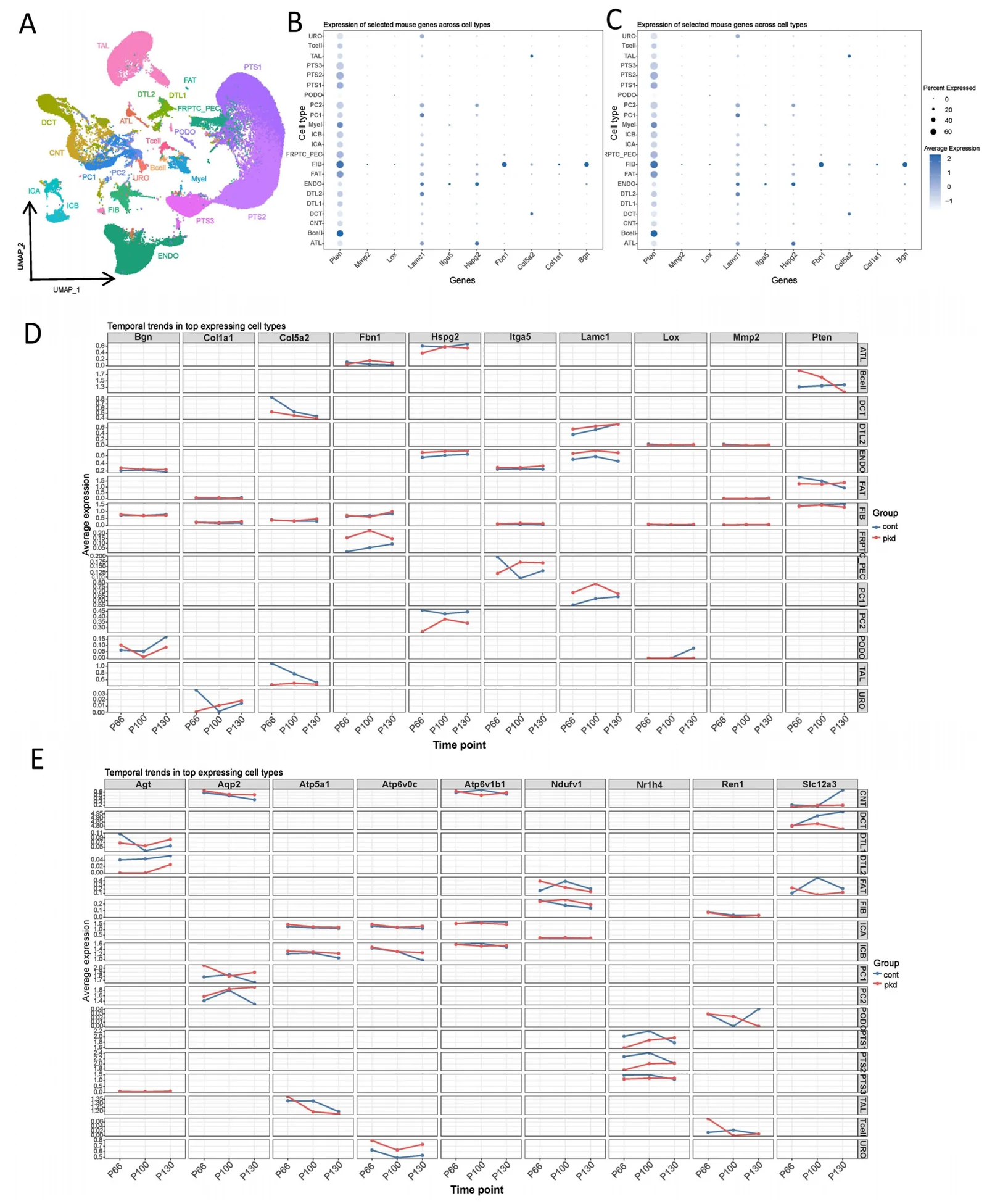
Single-cell validation of bulk-derived candidate modules. (**A**) UMAP visualization of annotated renal epithelial, stromal, vascular, and immune cell populations in the *Pkd1* time-course single-cell dataset. (**B**) Dot plot showing the expression of Cluster 3 candidate genes across cell types. Dot size indicates the percentage of expressing cells, and color indicates scaled average expression. (**C**) Dot plot showing the expression of Cluster 5 candidate genes across cell types. (**D**) Cell-type- and time-point-resolved expression trends of Cluster 3 genes across the *Pkd1* time course. (**E**) Cell-type- and time-point-resolved expression trends of Cluster 5 genes, validating directional consistency between bulk cyst-size-associated changes and single-cell disease progression.

The *Pkd1* time-course single-cell dataset identified major renal epithelial, stromal, vascular, and immune compartments including proximal tubule segments, loop-of-Henle populations, distal convoluted tubule, connecting tubule, collecting duct principal and intercalated cells, podocytes, fibroblasts, endothelial cells, B cells, T cells, and myeloid cells (Figure 5A). This annotation provided the cellular framework for interpreting the bulk-derived candidate genes.

Mapping of candidate genes revealed divergent cellular localization patterns for the two temporal modules (Figure 5B,C). ECM-remodeling genes were enriched mainly in fibroblast/stromal compartments, with selected genes also detected in endothelial or epithelial subsets. In contrast, tubular transport/metabolic genes showed nephron-segment-restricted epithelial expression: *Slc12a3* was enriched in distal convoluted tubule cells, *Aqp2* in collecting duct principal cells, and *Atp6v1b1* and *Atp6v0c* in intercalated-cell-associated compartments and mitochondrial genes across metabolically active epithelial populations.

Stage-stratified single-cell analyses showed consistent expression trends between human bulk cyst-size profiles and the murine *Pkd1* disease model (Figure 5D,E). Genes that increased with cyst enlargement in bulk tissue generally displayed increased expression in stromal/fibroblast compartments during *Pkd1* disease progression, whereas genes that decreased with cyst enlargement showed reduced epithelial expression over time. This cross-dataset consistency supports the interpretation that the bulk-derived signals represent biologically coherent, cell-type-resolved progression programs.

### 3.5. AUCell Analysis Reveals Cell-Type-Specific Pathway Rewiring

**Figure 6:**
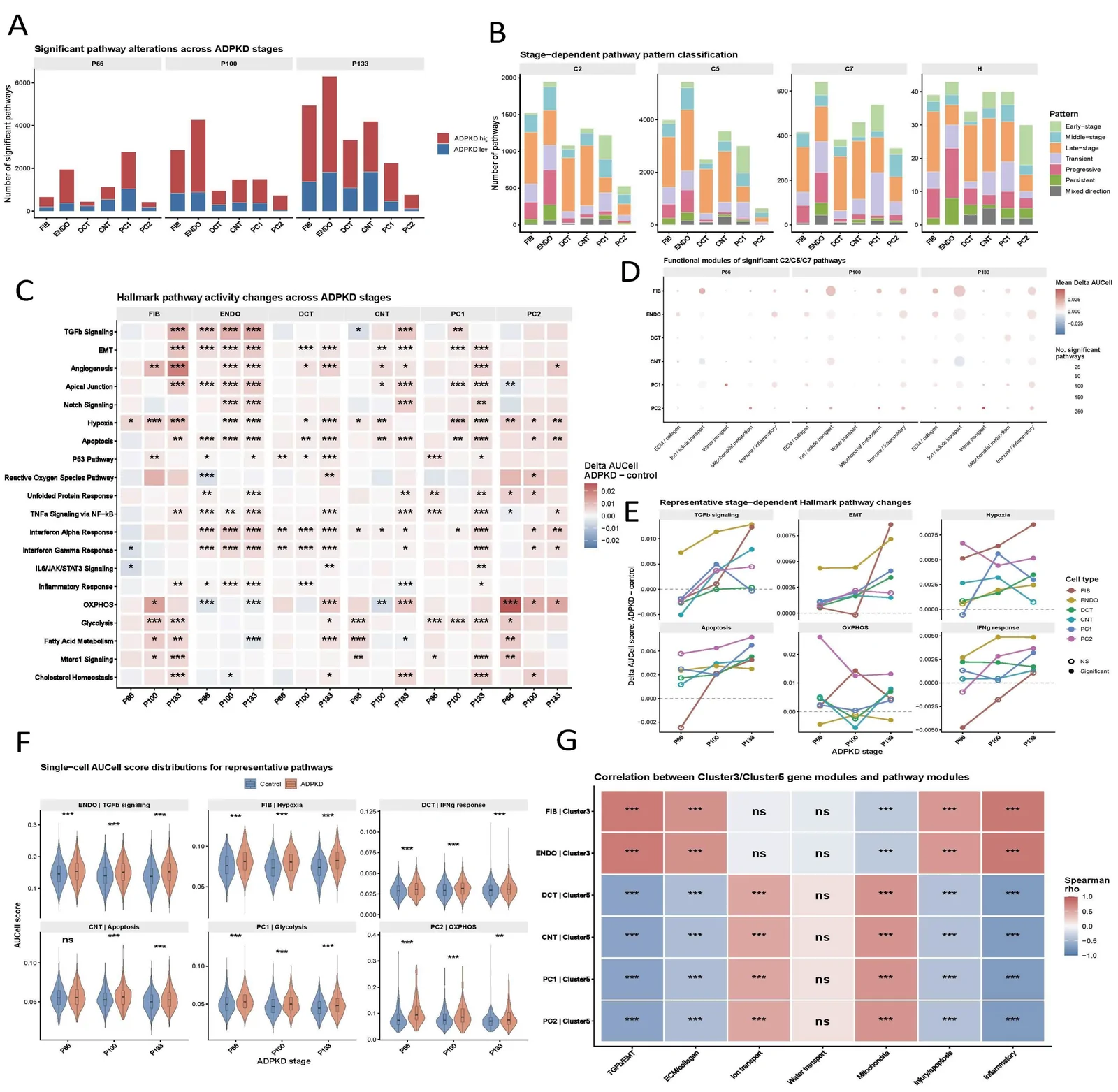
Cell-type-specific pathway scoring in the single-cell dataset. (**A**) Number of significantly altered AUCell pathway scores across cell types and disease stages. (**B**) Classification of pathway temporal patterns, including early, middle, late, transient, progressive, sustained, and bidirectional programs. (**C**) Heatmap summarizing representative pathway activities across selected cell-type/time-point combinations(* *p* < 0.05, ** *p* < 0.01, *** *p* < 0.001). (**D**) Module-level summary of fibrosis, hypoxia, inflammatory, apoptotic, epithelial–mesenchymal transition, and interferon-related pathway activation.The size of the bubbles represents the number of significant pathways. (**E**) Trajectory-style visualization of pathway activity changes across ordered *Pkd1* disease stages. (**F**) Violin and density plots comparing representative AUCell scores between the control and *Pkd1*-deficient groups (** *p* < 0.01, *** *p* < 0.001 and ns, not significant). (**G**) Correlation analysis linking bulk-derived ECM and tubular modules to cell-type-specific pathway signatures (*** *p* < 0.001 and ns, not significant).

AUCell pathway scoring showed that pathway remodeling was stage- and cell-type-dependent rather than uniform across the kidney (Supplementary Table S2). Significant pathway counts increased across disease stages and were prominent in epithelial compartments at later stages (Figure 6A). Pattern classification categorized pathway programs as early-stage, middle-stage, late-stage, transient, progressive, sustained, or bidirectional (Figure 6B), indicating that distinct maladaptive cell states emerge across renal cell types over disease progression. Heatmaps, module summaries, trajectory plots, and violin/density plots showed activation of fibrosis, hypoxia, inflammatory, apoptotic, epithelial–mesenchymal transition, and interferon-related pathways, together with the attenuation of oxidative phosphorylation and fatty-acid metabolic programs in selected tubular compartments (Figure 6C–F; Supplementary Figure S2). These pathway-level changes paralleled the bulk-derived Cluster 3 and Cluster 5 modules: the upregulated ECM module aligned with stromal/inflammatory pathway activation, whereas the downregulated tubular module aligned with reduced metabolic and epithelial homeostasis signatures.

Module-correlation analysis further connected the bulk temporal modules to cell-type-specific pathway activities (Figure 6G). Upregulated ECM genes correlated with stromal injury and inflammatory pathway scores, whereas downregulated tubular transport and metabolic genes correlated with mitochondrial function and epithelial identity signature. These findings suggest that ADPKD progression involves coordinated pathway rewiring across epithelial, immune, and stromal compartments rather than a single-cell-type-restricted response.

### 3.6. Ligand–Receptor Inference Suggests Altered Immune–Parenchymal Communication

**Figure 7:**
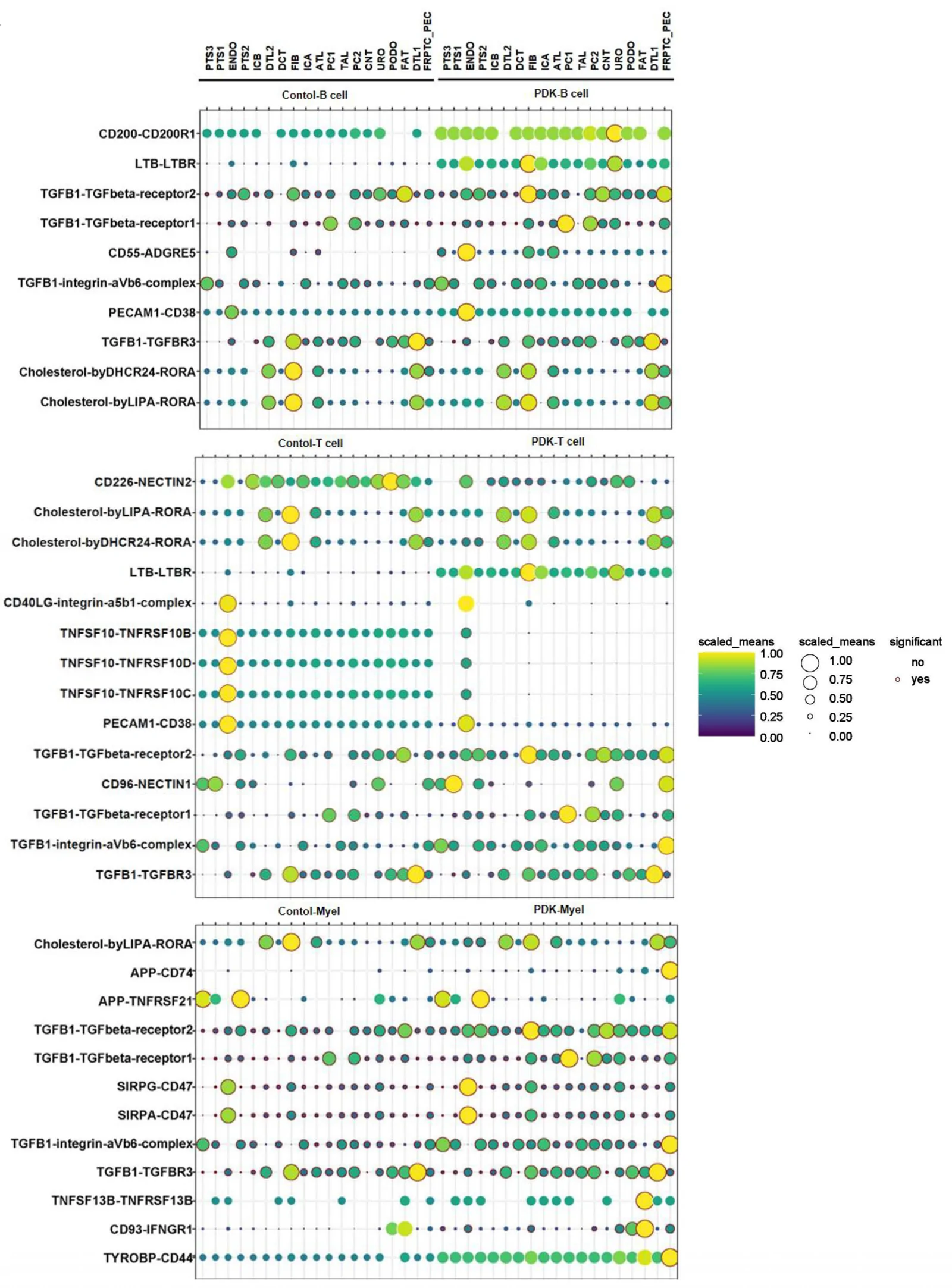
Cell–cell communication between immune cells and renal parenchymal/stromal compartments. Dot plot of significant ligand–receptor interactions inferred by CellPhoneDB for B cells, T cells and myeloid cells communicating with renal epithelial, endothelial, and stromal cell populations. The displayed interactions include CD200–CD200R1, LTB–LTBR, TGFB1–receptor complexes, TGFB1–integrin-aVb6, PECAM1–CD38, CD226–NECTIN2, CD96–NECTIN1, CD40LG–integrin, TNFSF10–receptor, SIRPA/SIRPG–CD47, TNFSF13B–TNFRSF13B, CD93–IFNGR1, and TYROBP-CD44. Dot size represents interaction strength or mean expression, and color represents scaled mean expression.

Ligand–receptor inference identifies rewired intercellular signaling between immune cells and renal parenchymal cells in *Pkd1*-deficient kidneys, as assessed by CellPhoneDB (Figure 7). B-cell-related interactions included *CD200–CD200R1, LTB–LTBR, TGFB1*–receptor complexes, TGFB1–integrin-αVβ6, PECAM1–CD38, and cholesterol-associated receptor axes. T-cell-related communication involved CD226–NECTIN2, CD96–NECTIN1, CD40LG–integrin, TNFSF10–receptor, PECAM1–CD38, and TGFB1-related interactions. Myeloid-cell interactions included TGFB1–receptor complexes, SIRPA/SIRPG–CD47, TNFSF13B–TNFRSF13B, CD93–IFNGR1, and TYROBP–CD44.

These inferred interactions are best interpreted as hypothesis-generating signals, but they were coherent with the immune deconvolution and single-cell localization results. Profibrotic *TGFB1* signaling may bridge immune cells to fibroblast and stromal activation; CD47, CD200, and PECAM1 interactions may mediate immune regulation and adhesion-dependent signaling; and TNF superfamily signaling may sustain inflammatory responses. Thus, ligand–receptor inference supports a model in which immune cells communicate with fibroblast, endothelial, and tubular epithelial compartments during ADPKD progression.

## 4. Discussion

This study supports a staged, multicellular model of ADPKD progression in which cyst enlargement is coupled to ECM remodeling, immune activation, and loss of tubular transport/metabolic identity. A central strength of the analysis is that the same directional programs were observed across complementary data layers: genes that increased or decreased with cyst size in human bulk transcriptomes showed plausible cellular localization and temporal behavior in the single-cell time-course dataset. This agreement reduces the likelihood that the findings reflect isolated platform-specific noise and instead supports the presence of reproducible biological programs associated with cyst progression [3,4].

The progressively upregulated module was dominated by ECM organization, collagen-containing matrix, adhesion, and matrix-remodeling terms. This pattern is biologically meaningful because ECM remodeling in ADPKD may not merely represent a passive consequence of advanced scarring. Expansion of collagen-rich matrix, basement-membrane remodeling, matrix protease activity, and LOX-mediated crosslinking can change tissue stiffness, ligand availability, and pericystic architecture. These changes may modify integrin signaling, epithelial polarity, mechanotransduction, fibroblast activation, and macrophage recruitment. Hub genes such as *COL1A1, COL5A2, FBN1, BGN, LAMC1, HSPG2, MMP2, LOX* and *ITGA5* therefore represent more than histological fibrosis markers; together, they characterize a matrix-remodeled niche that may actively reinforce cyst expansion and interstitial remodeling [8,9,27,28,29,30,31].

The localization of ECM-associated genes to fibroblast and stromal compartments provides an important cell-type-level interpretation of the bulk signal. In cystic kidneys, bulk transcriptomic changes can be driven either by altered expression within resident cells or by changes in tissue composition. The single-cell mapping suggests that the Cluster 3 program reflects increased matrix-related gene expression within fibroblast/stromal cells. This is consistent with kidney-fibrosis atlases showing that activated fibroblast and myofibroblast states organize ECM deposition, inflammatory signaling, and vascular remodeling [27,32]. In ADPKD, such stromal activation may be particularly consequential because expanding cysts impose mechanical distortion, local hypoxia, and altered epithelial–stromal contact zones. The convergence of *LOX, ITGA5, MMP2, HSPG2*, and collagen-family genes further suggests that matrix stiffness, proteoglycan remodeling, and integrin-dependent signaling may operate as linked components rather than as separate downstream changes.

The progressively downregulated module highlighted a different but complementary process: erosion of differentiated tubular epithelial function. Cluster 5 genes encoded solute transporters, proton-transport machinery, mitochondrial components, and nephron-segment identity markers including *SLC12A3, AQP2, ATP5A1, ATP6V0C, ATP6V1B1, NDUFV1, REN*, and *AGT*. Their decline suggests that cyst progression is accompanied by epithelial functional dedifferentiation rather than by matrix deposition alone. This finding aligns with current views that ADPKD involves metabolic reprogramming, mitochondrial dysfunction, altered oxidative phosphorylation, and impaired fatty-acid metabolism [16,33,34]. Importantly, reduced tubular transport and metabolic gene expression may contribute to disease propagation by weakening epithelial energy homeostasis, disrupting ion and water handling, and increasing susceptibility to stress-induced maladaptive repair.

The immune results add a third layer to this model. xCell deconvolution linked ECM hub genes to macrophage- and mast-cell-associated signals, and ligand–receptor inference suggested altered communication through TGFB1-related, CD47-related, TNF-superfamily, CD200–CD200R1, PECAM1–CD38, and integrin-associated axes. These observations are consistent with recent ADPKD studies emphasizing that immune cells participate in cystic microenvironment remodeling rather than simply accumulating as bystanders [5,7]. M2-like macrophage-associated signatures are particularly relevant because they may connect chronic inflammation with fibroblast activation and matrix deposition. Meanwhile, CD47- and CD200-related interactions may reflect immune-regulatory circuits that shape myeloid activation, immune persistence, and epithelial immune evasion within the cystic niche.

The ligand–receptor findings should be interpreted cautiously but are biologically informative. CellPhoneDB does not prove physical contact or signaling flux; it identifies expression-compatible communication axes. Nevertheless, when these inferred interactions are considered together with immune deconvolution and single-cell localization, they support a coherent model in which immune cells, fibroblasts, endothelial cells, and tubular epithelial cells form a feed-forward circuit. In this circuit, epithelial stress and cyst expansion may promote inflammatory recruitment; immune cells may release profibrotic and immune-regulatory signals; fibroblast activation may increase matrix stiffness and alter tissue architecture; and remodeled matrix may further impair epithelial polarity and metabolic competence. Similar integrated bioinformatic, transcriptomic, and single-cell strategies have been used in recent kidney injury and immune kidney disease studies, supporting the value of cross-dataset prioritization while underscoring the need for experimental validation [35,36,37,38,39,40,41].

The AUCell results further indicate that pathway remodeling is stage- and cell-type-specific. The increase in significant pathway counts at later disease stages, together with activation of fibrosis, hypoxia, apoptosis, epithelial–mesenchymal transition, and interferon-related programs, suggests that ADPKD progression is not a uniform kidney-wide stress response. Instead, distinct cell populations appear to enter different maladaptive states over time. This point is important for therapeutic implication: interventions aimed only at cyst epithelial proliferation may not fully address the stromal, immune, and metabolic programs that become established during progression. A combined strategy that considers epithelial transport/metabolic preservation, matrix remodeling, and immune–stromal communication may therefore provide broader mechanistic insight than a single-axis model.

From a translational perspective, the candidate genes identified here should be viewed as prioritization markers for experimental validation rather than immediate therapeutic targets. ECM genes such as LOX, ITGA5, and MMP2 may point to matrix crosslinking, integrin signaling, and protease-dependent remodeling as testable mechanisms. Tubular genes such as *AQP2, SLC12A3*, and *ATP6V1B1* may serve as readouts of segment-specific epithelial identity loss. Immune communication axes involving TGFB1, CD47, CD200, and TNF-superfamily ligands may help nominate immune–stromal interactions for spatial validation. The most informative next experiments would combine spatial transcriptomics, multiplex immunostaining, and protein-level validation to determine whether these signals colocalize around expanding cysts, fibrotic septa, and remodeled vessels.

Several limitations should be acknowledged. First, the analysis relies on public datasets and requires validation in independent human ADPKD tissues. However, clinically indicated renal biopsy specimens from ADPKD patients are rare because diagnosis is usually established by imaging and genetic assessment, and biopsy is not routinely performed in typical cystic kidneys because of limited clinical necessity and procedural risk. This clinical scarcity currently limits immediate validation using local human ADPKD biopsy samples. Second, GSE7869 provides valuable cyst-size information but is microarray-based and limited in sample size, which may reduce statistical power for weaker but biologically relevant signals. Third, immune deconvolution, enrichment analysis, AUCell scoring, and ligand–receptor inference are hypothesis-generating computational approaches and should be followed by spatial, protein-level, and functional validation. Finally, the mouse and human genomes are not identical, and animal models cannot fully replicate human genetic diversity or environmental exposures (e.g., diet and lifestyle); Human samples were predominantly from advanced stages, and cyst size in the human dataset is not fully equivalent to chronological disease progression in the animal model.

Future work should focus on testing whether the inferred modules are spatially organized and functionally active in cystic tissue. Spatial transcriptomics or multiplex immunostaining could determine whether ECM hub genes localize to pericystic fibrotic septa, vascular niches, or fibroblast-rich regions. Protein-level validation of *COL1A1, COL5A2, LOX, ITGA5, MMP2, SLC12A3, AQP2, ATP5A1*, and *ATP6V1B1* would strengthen translational relevance. Perturbation experiments targeting matrix crosslinking, integrin signaling, macrophage-fibroblast communication or tubularmitochondrial metabolism will be needed to distinguish causal drivers from progression biomarkers. On this basis, human ADPKD kidney organoid models will be used to verify the regulatory functions of core genes on cyst pathological phenotypes in a human-derived system. *Pkd1* conditional knockout mouse models will be employed for in vivo target intervention experiments to test the therapeutic efficacy of key pathways in slowing cyst progression. In addition, an ADPKD clinical follow-up cohort will be established to systematically analyze the correlations between the expression levels of core hub genes and patients’ cyst burden, renal function decline, and long-term prognosis, further evaluating their clinical potential as novel diagnostic and prognostic biomarkers.

## 5. Conclusions

In conclusion, integrated analysis of cyst-size-stratified human transcriptomes and cell-type-resolved single-cell data identified two opposing progression programs in ADPKD: a progressively upregulated ECM/stromal module centered on *COL1A1, COL5A2, FBN1, BGN, LAMC1, HSPG2, MMP2, LOX*, and *ITGA5*, and a progressively downregulated tubular transport/metabolic module centered on *SLC12A3, AQP2, ATP5A1, ATP6V0C, ATP6V1B1, NDUFV1, REN*, and *AGT*. Immune deconvolution, AUCell scoring, and ligand–receptor inference further linked these modules to macrophage-associated remodeling, fibrosis/hypoxia/inflammatory pathway activation, and immune–parenchymal communication axes involving TGFB1, CD47, CD200–CD200R1, PECAM1–CD38, integrin-related signaling, and TNF-superfamily interactions. These findings provide a stage-resolved molecular framework for ADPKD progression and nominate specific ECM, tubular metabolic, and immune–stromal candidates for future spatial validation and functional testing.

## Data Availability

The datasets analyzed in this study are publicly available from the Gene Expression Omnibus including GSE7869 and GSE268494. Processed result tables are provided as Supplementary Materials or are available from the corresponding author upon reasonable request.

## Supplementary Materials

The following supporting information can be downloaded at: https://www.mdpi.com/article/10.3390/biology15211939/s1, Figure S1:Expression patterns of prioritized hub genes across ADPKD cyst-size groups; Figure S2: Supplementary AUCell analysis of cell-type-specific pathway remodeling in the *Pkd1* single-cell dataset. Table S1: Summary of differentially expressed genes (DEGs) identified across small-, middle-, and large-cyst groups. Table S2: Supplementary AUCell analysis of stage- and cell-type-specific pathway remodeling in the *Pkd1* single-cell dataset.
