# Supplementary material for: Integrated Bulk Transcriptomic and Single-Cell Analyses Reveal Extracellular-Matrix Remodeling and Tubular Metabolic Failure in ADPKD Progression
Source: Biology (Basel). 2026 Oct 25;15(21):1939. doi: 10.3390/biology15211939 (PMC13762317; doi:10.3390/biology15211939)
Supplement: Supplementary file 1 [file biology-15-01939-s001.zip › Supplementary_Information.pdf]

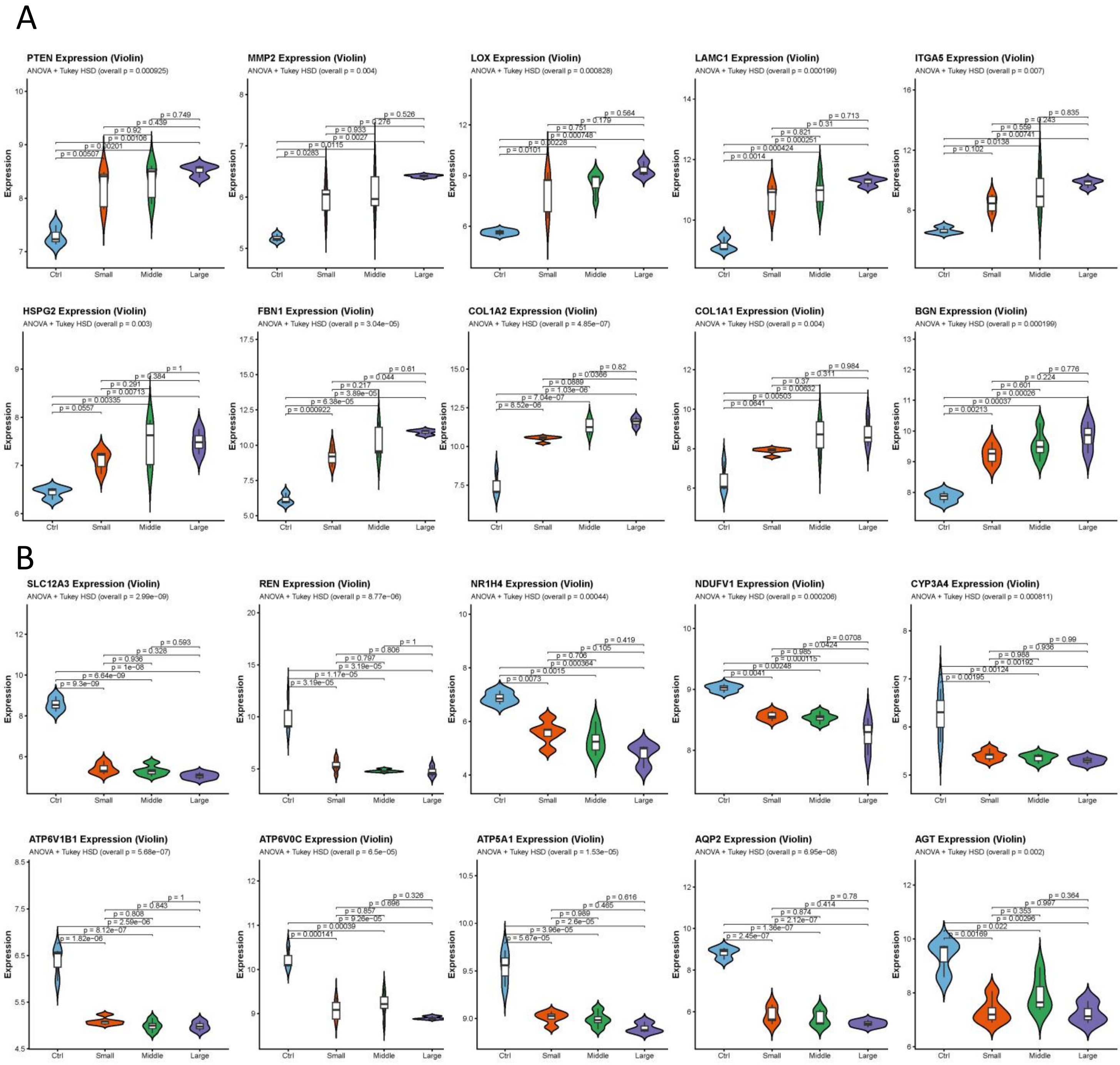
A
PTEN Expression (Violin)
ANOVA + Tukey HSD (overall p = 0.000925)
MMP2 Expression (Violin)
ANOVA + Tukey HSD (overall p = 0.004)
LOX Expression (Violin)
ANOVA + Tukey HSD (overall p = 0.000828)
LAMC1 Expression (Violin)
ANOVA + Tukey HSD (overall p = 0.000199)
ITGA5 Expression (Violin)
ANOVA + Tukey HSD (overall p = 0.007)
HSPG2 Expression (Violin)
ANOVA + Tukey HSD (overall p = 0.003)
FBN1 Expression (Violin)
ANOVA + Tukey HSD (overall p = 3.04e-05)
COL1A2 Expression (Violin)
ANOVA + Tukey HSD (overall p = 4.85e-07)
COL1A1 Expression (Violin)
ANOVA + Tukey HSD (overall p = 0.004)
BGN Expression (Violin)
ANOVA + Tukey HSD (overall p = 0.000199)
B
SLC12A3 Expression (Violin)
ANOVA + Tukey HSD (overall p = 2.99e-09)
REN Expression (Violin)
ANOVA + Tukey HSD (overall p = 8.77e-06)
NR1H4 Expression (Violin)
ANOVA + Tukey HSD (overall p = 0.00044)
NDUFV1 Expression (Violin)
ANOVA + Tukey HSD (overall p = 0.000206)
CYP3A4 Expression (Violin)
ANOVA + Tukey HSD (overall p = 0.000811)
ATP6V1B1 Expression (Violin)
ANOVA + Tukey HSD (overall p = 5.68e-07)
ATP6V0C Expression (Violin)
ANOVA + Tukey HSD (overall p = 6.5e-05)
ATP5A1 Expression (Violin)
ANOVA + Tukey HSD (overall p = 1.53e-05)
AQP2 Expression (Violin)
ANOVA + Tukey HSD (overall p = 6.95e-08)
AGT Expression (Violin)
ANOVA + Tukey HSD (overall p = 0.002)
Expression
Ctrl
Small
Middle
Large

Supplementary Figure S1. Expression patterns of prioritized hub genes across ADPKD cyst-size groups. Expression distributions of candidate genes derived from the progressively upregulated Cluster 3 and progressively downregulated Cluster 5 modules in GSE7869. (A) Expression patterns of Cluster 3 extracellular-matrix and adhesion-related hub genes across control, small-cyst, middle-cyst and large-cyst samples. Representative genes include COL1A1, COL5A2, FBN1, BGN, LAMC1, HSPG2, MMP2, LOX and ITGA5. These genes generally showed increased expression with increasing cyst size, supporting progressive extracellular-matrix remodeling during ADPKD progression. (B) Expression patterns of Cluster 5 tubular transport and metabolic hub genes across the same cyst-size groups. Representative genes include SLC12A3, REN, NDUFV1, ATP6V1B1, ATP6V0C, ATP5A1, AQP2 and AGT. These genes generally showed reduced expression with increasing cyst size, consistent with progressive loss of differentiated tubular epithelial transport and metabolic identity. Data are shown as normalized expression values. Statistical comparisons were performed across cyst-size groups as described in the Methods.

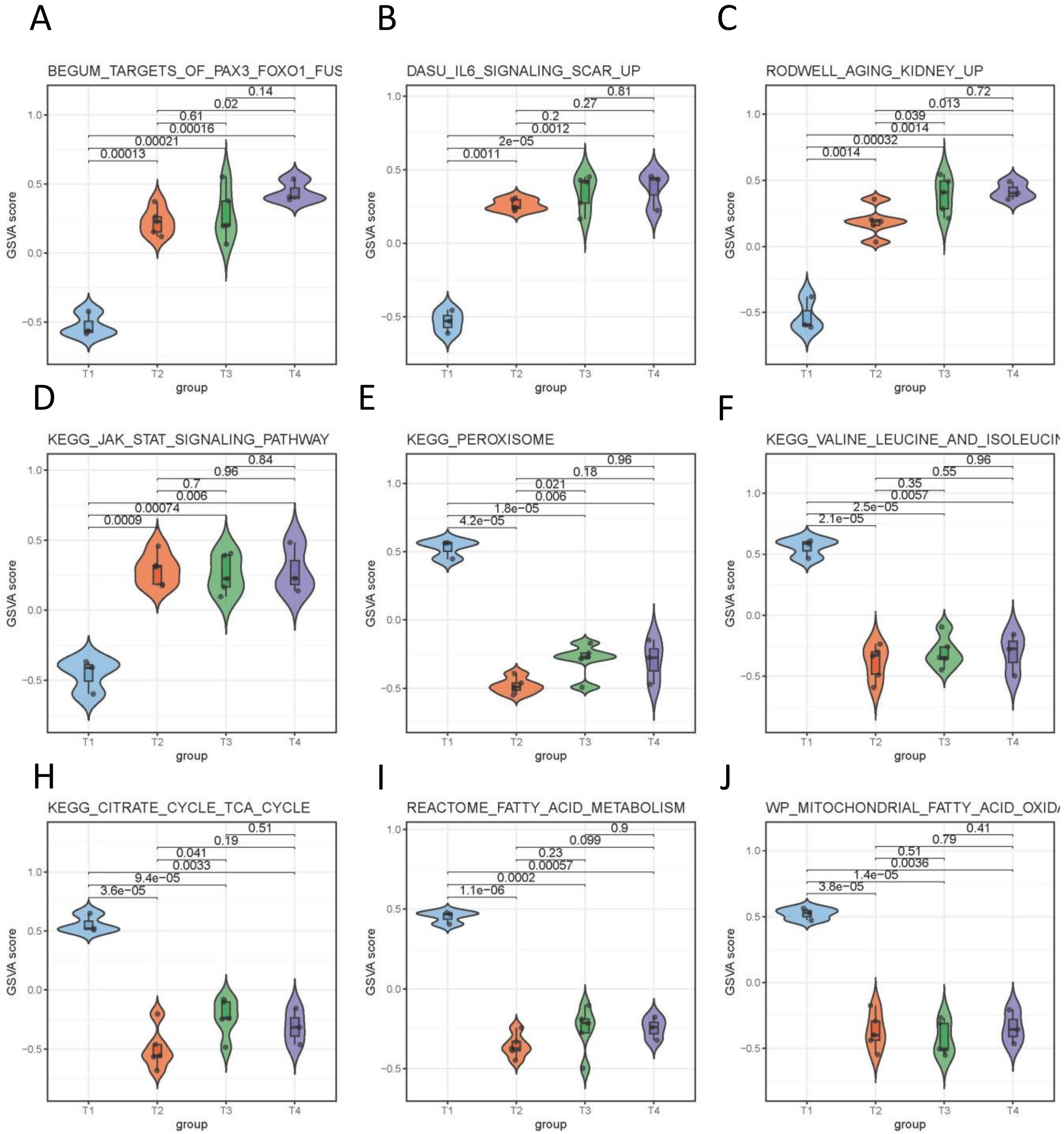

A
BEGUM_TARGETS_OF_PAX3_FOXO1_FUS
0.14
0.02
0.61
0.00016
0.00021
0.00013
B
DASU_IL6_SIGNALING_SCAR_UP
0.81
0.27
0.2
0.0012
2e−05
0.0011
C
RODWELL_AGING_KIDNEY_UP
0.72
0.013
0.039
0.0014
0.00032
0.0014
D
KEGG_JAK_STAT_SIGNALING_PATHWAY
0.84
0.96
0.7
0.006
0.00074
0.0009
E
KEGG_PEROXISOME
0.96
0.18
0.021
0.006
1.8e−05
4.2e−05
F
KEGG_VALINE_LEUCINE_AND_ISOLEUCIN
0.96
0.55
0.35
0.0057
2.5e−05
2.1e−05
H
KEGG_CITRATE_CYCLE_TCA_CYCLE
0.51
0.19
0.041
0.0033
9.4e−05
3.6e−05
I
REACTOME_FATTY_ACID_METABOLISM
0.9
0.099
0.23
0.00057
0.0002
1.1e−06
J
WP_MITOCHONDRIAL_FATTY_ACID_OXID
0.41
0.79
0.51
0.0036
1.4e−05
3.8e−05
GSVA score
group
T1
T2
T3
T4

Supplementary Figure S2. Supplementary AUCell analysis of cell-type-specific pathway remodeling in the Pkd1 single-cell dataset. (A) Heatmap showing representative AUCell pathway activities across annotated renal epithelial, stromal, vascular and immune cell populations at different disease stages. Pathway scores highlight coordinated activation of fibrosis, hypoxia, inflammatory, apoptotic, epithelial-mesenchymal transition and interferon-related programs. (B) Module-level summary of selected pathway categories, showing increased injury- and remodeling-associated pathway activity in specific cell populations during Pkd1 disease progression. (C) Stage-resolved trajectory-style visualization of pathway activity changes across ordered Pkd1 disease stages, illustrating temporal activation or attenuation of major biological programs. (D) Violin and density plots comparing representative AUCell scores between control and Pkd1-deficient groups, including fibrosis/inflammation-associated pathways and metabolic pathways such as oxidative phosphorylation and fatty-acid metabolism. Together, these analyses support cell-type- and stage-specific pathway rewiring during ADPKD progression and complement the bulk-derived Cluster 3 extracellular-matrix module and Cluster 5 tubular metabolic module.
